# Proteomic Analysis of Two Weight Classes of Mule Duck “*foie gras*” at the End of an Overfeeding Period

**DOI:** 10.3389/fphys.2020.569329

**Published:** 2020-09-17

**Authors:** Bara Lo, Nathalie Marty-Gasset, Carole Pichereaux, Céline Bravo, Hélène Manse, Renaud Domitile, Hervé Rémignon

**Affiliations:** ^1^Institut National de Recherche Pour l’Agriculture, l’Alimentation et l’Environnement, Ecole Nationale Vétérinaire de Toulouse, Université de Toulouse, GENétique PHYsiologie et Systèmes d’Elevage, Castanet-Tolosan, France; ^2^Centre National de la Recherche Scientifique, Fédération de Recherche (FR3450), Agrobiosciences, Interactions et Biodiversité, Toulouse, France; ^3^Centre National de la Recherche Scientifique, Université de Toulouse – UPS, Institut de Pharmacologie et Biologie Structurale, Toulouse, France; ^4^Idena, Sautron, France

**Keywords:** “*foie gras”*, proteomic, liver weight, mule duck, oxidative stress, cell death, cell structure

## Abstract

The weight of the liver is one of the important selection criteria in the quality of “foie gras”. This factor is highly variable despite the fact that individuals are reared, overfed and slaughtered in the same way. In this study, we performed an analysis of the proteome profile of two weight classes of light (between 550 and 599 g) and heavy (more than 700 g) livers. For the analysis of the proteic extracts, a liquid chromatographic analysis coupled with mass spectrometry was carried out. In low-weight livers, aerobic energy metabolism, protein metabolism and lipid metabolism oriented toward export and beta-oxidation were overexpressed. On the contrary, high weight livers were characterized by anaerobic energy metabolism and a more active protein catabolism associated with cell apoptosis and reorganization of the cell structure.

## Introduction

The production of “foie gras” is a tradition that has crossed the ages since the ancient Egyptians. To obtain this product, palmipeds are overfed twice a day for a minimum period of 10 days with a hypercaloric diet, based on corn supplemented or not with a commercial premix (Bonnefont et al., 2019), to induce a steatosis of the liver. Indeed, the corn is mainly composed of starch (about 64%, Souci et al., 2000) acting as the precursor of *de novo* lipogenesis which takes place mainly in the liver in birds. In addition, the reabsorption of circulating lipids allows the liver to also become the main place of fat storage during this process (Hermier et al., 1999). In ducks, different liver weights, within a range of 500 to more than 700 g, are generally obtained according to rearing and overfeeding conditions (Théron, 2011; Awde et al., 2013; Rémignon et al., 2018). Proteins play a key role in the metabolism of the liver during the development of the fatty liver induced by overfeeding although they represent less than 8% of the total fresh matter (Théron, 2011; Bax et al., 2012) at the end of the overfeeding period. During the development of this hepatic steatosis, several studies have reported differences in the expression levels of genes involved in different cellular pathways such as glycolysis, biosynthesis, transport, storage and oxidation of lipids and cytoskeletal modifications (Zhu et al., 2011; Bax et al., 2012). According to Bax et al. (2012), overfeeding can be separated into two stages with a first one characterized by a high level of anabolism to face overfeeding and a second one aimed at maintaining cell homeostasis.

Studies done at the end of the overfeeding period showed a huge increase in protein and lipid metabolisms but also in oxidative stress mechanisms and even cell death by apoptosis (Bénard and Labie, 1998; Théron, 2011; Rémignon et al., 2018; Lo et al., 2020). Anavi et al. (2017) reported that the hepatic tissues must have an effective delivery of oxygen to support the high intensity of its metabolism. However, lack of oxygen deliverance to the tissues causes hypoxia. According to Rankin et al. (2009), hypoxia leads to decrease the expression of lipogenic genes and increases the lipid storage capacity.

Although conditions of rearing and overfeeding are set to be the same for all the animals within a given flock, it is not uncommon for liver weights to finally largely differ. Such variability in the final weight of the livers questions professionals because it represents difficulties in managing the production but it also alters the sensorial properties of the final product, i.e., the “foie gras.” To better understand the development of the hepatic steatosis induced by an overfeeding, the present study aimed to measure the influence of the final liver weight on the expression of proteins in ducks of the same breed, reared and overfed under the same conditions. To have an overview of the implication of proteins in this process, we performed a comparison of proteomic profiles by liquid chromatography coupled to mass spectrometry between two groups of ducks presenting different liver weights at the end of the overfeeding period. Those variant proteins were then assigned to their respective metabolic pathways in order to clarify which of them could be considered as characteristics of each liver weight classes.

## Materials and Methods

### Animals and Samples

A flock of about 1000 male mule ducks (*Caïrina moschata x Anas platyrhynchos*) was reared for 12 weeks according to standard commercial rules. Then, according to a standard overfeeding program, birds were overfed with 20 meals (twice a day during 10 days) with increasing amounts of a corn diet ranging from 230 g/bird on day 1 to 480 g/bird on day 10. Finally, each duck ate around 8.5–8.7 kg of corn during all the overfeeding period. Approximately 11 h after the last meal, birds were conventionally slaughtered in a commercial slaughter house. After evisceration, livers were automatically weighed and 60 of them were randomly sampled to create two experimental groups: 30 livers weighing between 550 and 599 g (low weight liver or LWL group) and 30 livers with a weight over than 700 g (high weight liver or HWL group). From those 60 livers, 50 g were collected 20 min post-mortem from the median lobe and directly frozen in liquid nitrogen before storage at −80°C. The rest of the livers was cooled to 4°C. Then, Near Infra-Red Spectroscopy (NIRS) measurements were performed on six independent points of the surface of the liver with a spectrometer (Labspec^®^ 5000 Pro, ASD, Inc., Boulder, CO, United States) to predict the gross biochemical characteristics of livers.

### Biochemical Analysis

For all samples, the gross biochemical (i.e., dry matter, total lipids, and total nitrogen) contents were determined from NIRS spectra according to the method described by Marie-Etancelin et al. (2014). 24 samples (12/groups) were then selected for being representatives of the whole 60 samples and further analyzed for proteomics. The total amount of proteins was determined by the formula: (% Proteins = % total nitrogen × 6.25).

### Proteomic Analysis

#### Proteins Extraction

Proteins were extracted from samples according to a modified protocol of Sayd et al. (2006). Briefly, a buffer containing 7M urea, 2M thiourea, 4% 3-[(3-cholamidopropyl) dimethylammonio]-1-propane sulfate (CHAPS) and 1% Dithiothreitol (DTT) was added to liver powder, in a ratio of 1:4 (w/v) at 4°C. Then, two homogenization series were performed in Retsch MM400 (Haan, Germany) at 30 Hz for 30 min at 4°C followed by a centrifugation at 1000 *g* at 4°C for 10 min. Then, the fat cake formed at the surface of the extract was removed and a second centrifugation was performed under the same conditions. Finally, the supernatant was collected and its proteins concentration was measured according to Bradford (1976).

#### Sample Preparation for Proteomics Analysis

Thirty μl of proteins samples were reduced (Laemmli, 1970) in a Laemmli’s buffer containing 25 mM dithiothreitol (DTT) at 95°C for 5 min and cysteines were alkylated by the addition of 90 mM iodoacetamide for 30 min at room temperature. 75 μg of proteins were then loaded onto a 12% SDS-polyacrylamide gel and subjected to a short electrophoresis. After InstantBlue (Expedeon, United Kingdom) staining of the gel, the gel band was excised, washed twice with 50 mM ammonium bicarbonate-acetonitrile (1:1, v:v) and then washed once with acetonitrile. Proteins were in-gel digested by the addition of 60 μL of a solution of modified sequencing grade trypsin in 25 mM ammonium bicarbonate (20 ng/μL, sequence grade, Promega, Charbonnières-les-Bains, France). The mixture was incubated at 37°C overnight. The resulting peptides were then extracted from the gel by one round of incubation (15 min, 37°C) in 1% formic acid–acetonitrile (60–40%) and two rounds of incubation (15 min each, 37°C) in 1% formic acid–acetonitrile (1:1). The extracted fractions were air-dried. Finally, tryptic peptides were resuspended in 14 μl of 2% acetonitrile and 0.05% trifluoroacetic acid for further MS analysis.

#### NanoLC-MS/MS Analysis

Peptide mixtures were analyzed by nanoLC-MS/MS using a nanoRS UHPLC system (Dionex, Amsterdam, Netherlands) coupled to an LTQ-Orbitrap Velos mass spectrometer (Thermo Fisher Scientific, Bremen, Germany). Each biochemical replicate (three per conditions) samples was analyzed twice. Five microliters of each sample were loaded on a C18 pre-column (5 mm × 300 μm; Dionex) at 20 μL/min in 2% acetonitrile and 0.05% trifluoroacetic acid. After 5 min of desalting, the pre-column was switched online with the analytical C18 column (50 cm × 75 μm inner diameter; in-house packed with Reprosil C18) equilibrated in 95% of solvent A (5% acetonitrile + 0.2% formic acid in water) and 5% of solvent B (80% acetonitrile + 0.2% formic acid in water). Peptides were then eluted using a 5–50% gradient of solvent B for 105 min at a 300 nL/min flow rate. The LTQ-Orbitrap was operated in data-dependent acquisition mode with Xcalibur software. Survey scan MS spectra were acquired in the Orbitrap on the 300–2000 m/z range with the resolution set to a value of 60,000. The 20 most intense ion survey scans were selected for CID (collision-induced dissociation) fragmentation and the resulting fragments were analyzed in the linear trap (LTQ). Dynamic exclusion was used within 60 s to prevent repetitive selection of the same peptide.

#### Protein Identification and Quantification: Database Search and Data Analysis

Acquired MS and MS/MS data as Raw MS files were converted to the mzdb format and were processed with the mzdb-access library^1^ to generate peaklists. Data were searched with Mascot (version 2.6.1^2^) against a custom-made database containing *Anas platyrhynchos & Cairina moschata* entries from the UniProtKB database (Swiss-Prot/TrEmbl release 20190524, 35,568 entries).

The search included methionine oxidation as a variable modification and carbamidomethylation of cysteine as a fixed modification. Specificity of digestion was set for cleavages after lysine or arginine for trypsin-digested samples and only one missed cleavage was allowed. The mass tolerance was set to 6 ppm for the precursor ion. It was set to 0.8 Da for fragment ions in CID mode (detection in the ions trap). Validation of identifications was performed through a false-discovery rate set to 1% at protein and peptide-sequence match level determined by target-decoy search using the in-house-developed software Prolinesoftware version 1.6^3^ (Bouyssié et al., 2020). Raw MS signal extraction of identified peptides was performed with Proline across different samples. The mass spectrometry proteomics data has been submitted to the ProteomeXchange Consortium via the PRIDE partner repository with the dataset identifier PXD019866.

### Statistical Analysis

For all comparisons, statistical analysis was applied to abundance values. Statistical analyses were performed with the R software (version 3.6.3). First, Student/Wilcox mean comparison analysis was performed and a difference in expression was only considered to be significant only with a *p*-value < 0.05 with multiple testing correction false-discovery rate (FDR) and a mean normalized area ratio < 0.67 and > 1.5. Volcano plots were drawn to visualize significant protein abundance variations between the two studied groups. They represent log_10_ (*p*-value) according to the log_2_ (ratio). Then, a Partial Least Squares-Discriminant Analysis (PLS-DA) between the two studied groups was performed on significant proteins. Finally, a protein was considered as a discriminating one only if its Variable Importance in Projection (VIP) value was higher than 0.8.

### Gene Ontology (GO)

For all the proteins identified as variants, a gene ontology (GO) analysis was performed with the ProteINSIDE webservice (Kaspric et al., 2015) to identify the associated biologicals processes. Based on the fact that, in the used database (Canard_*Anas_Caïrina* 190524), the majority of the identified proteins are unreviewed proteins, all variant proteins were evaluated one by one and the ones not related to a function in the liver were deleted before GO analysis. The Human species was used for the GO analysis. For the GO enrichment analyses, only the different biological processes with a *p*-value < 10^–3^ and associated with at least two proteins of the list were considered. As the analysis was performed with a human model, after the GO analysis, all pathways not associated with the liver (viral process, brain development, heart development…) were considered irrelevant and not reported.

The experiments described here fully comply with legislation on research involving animal subjects according to the European Communities Council directive of November 24, 1986 (86/609/EEC). Investigators were certificated by the French governmental authority (agreement no. R-31-ENVT-F1-12) and Federation of European Laboratory Animal Science Associations (agreement no. F011/05) for carrying out those experiments.

## Results

### Biochemicals Analysis

**FIGURE 1:**
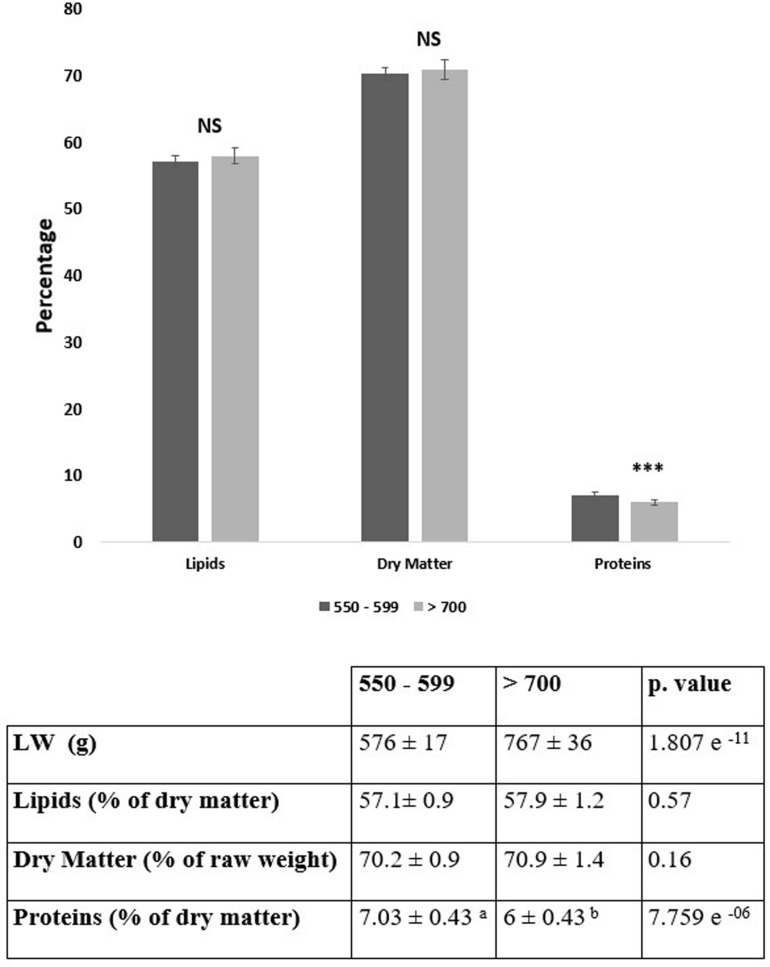
Liver weights (LW) and gross chemical composition (lipids, dry matter, proteins) of livers from the two studied groups (*n* = 12/groups), i.e., low weight livers (550–599 g) and high weight livers (>700 g). Values are means ± SD (*p ≤ 0.05, **p ≤ 0.01, ***p ≤ 0.001 and NS = not significant).

As expected, in the whole flock of about 1000 slaughtered male mule ducks, the average liver weight was 580 ± 87 g. Liver weights and total protein contents from the two experimental groups were largely different (*p*-value < 0.001, Figure 1). On the contrary, no significant differences (*p*-value > 0, 05) were observed for dry matter and total lipid contents between the two studied groups. These results are in accordance with observations made in previous studies (Rémignon et al., 2018; Bonnefont et al., 2019).

### Proteomic Analysis

**FIGURE 2:**
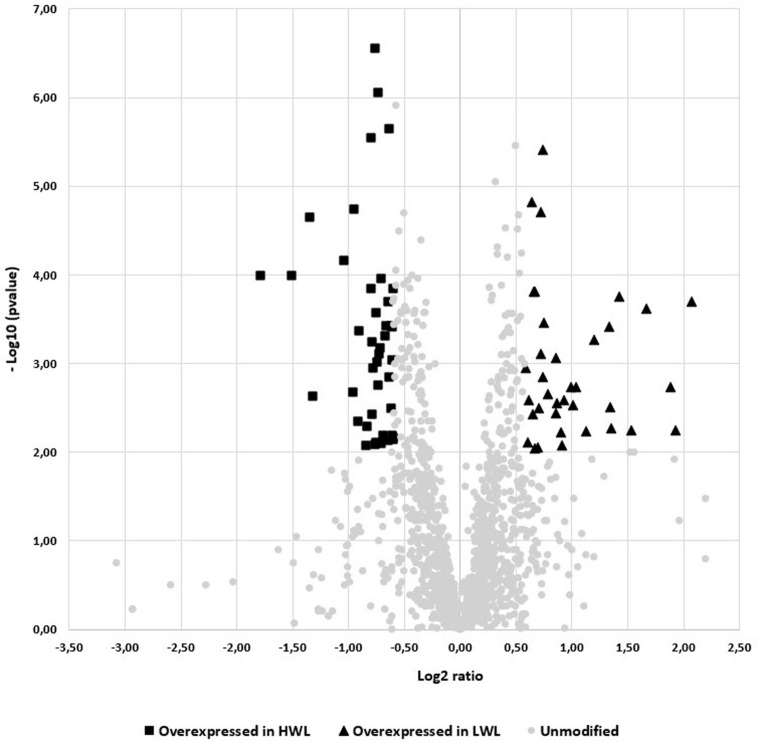
Volcano-plot of the proteins expressed in the two studied groups (*n* = 12/groups). Proteins with an unmodified level of expression were represented by circles, proteins overexpressed in the LWL group were represented by triangles and proteins overexpressed in the HWL group of livers were represented by squares.

The queries were carried out with the Mascot search engine using MS and MS/MS data from the UniProt sub-bank: Canard_*Anas_Caïrina* 190524 (32,568 sequences; 14,848,422 residues). As a result, 1444 quantified proteins were identified. Among them, only 235 proteins, exhibiting a *p*-value < 0.05 when comparing the two liver weight groups, were kept for the means comparison test. Considering that a significant difference (*p*-value < 0.05) in the level of expression of proteins did not necessarily mean a measurable effect at the phenotypic level, only proteins expression with a ratio of HWL/LWL more than 1.5 or less than 0.67 were considered because these threshold could possibly have a perceptible effect. Then, by using this significant expression ratio between the 2 groups, only 78 proteins were identified as pure variants. They represent 33% of the 235 proteins with a *p*-value < 0.05 when comparing the two liver weights groups. Among these proteins, more proteins (42) were overexpressed in the HWL group than in the LWL group (36 overexpressed proteins) (Figure 2).

The GO enrichment analysis of those variant proteins list allowed to identify 61 proteins involved in a biological process with a *p*-value < 10^–3^ and with at least two identified proteins per biological process: 33 of them were overexpressed in the low weight liver group (Table 1A) and 28 were overexpressed in the high weight liver group (Table 1B).

**TABLE 1:** List of proteins after GO enrichment analyses. **(A)** Biological process associated to overexpressed proteins in low weight livers (LWL). Ratio = intensity LWL/intensity HWL. **(B)** Biological process associated to overexpressed proteins in high weight livers (HWL). Ratio = intensity HWL/intensity LWL.

Accession	Symbol	Entrez gene name	Location	Type(s)	Gene_name	Pval	Ratio
**(A) Respiratory electron transport and ATP synthesis**							
U3J9G0	NDUFA4	NDUFA4 mitochondrial complex associated	Cytoplasm	Enzyme	NDUFA4	9,13E–04	1,52
R0LRI8	NDUFA2	NADH:ubiquinone oxidoreductase subunit A2	Cytoplasm	Enzyme	NDUFA2	4,22E–04	1,87
U3I6S2	NDUFB6	NADH:ubiquinone oxidoreductase subunit B6	Cytoplasm	Enzyme	NDUFB6	2,22E–05	2,54
R0M475	NDUFV1	NADH:ubiquinone oxidoreductase core subunit V1	Cytoplasm	Enzyme	Anapl_06614	5,80E–04	1,72
R0K870	NDUFA9	NADH:ubiquinone oxidoreductase subunit A9	Cytoplasm	Enzyme	Anapl_04036	3,71E–04	1,54
R0KC07	CHCHD2P9	Coiled-coil-helix-coiled-coil-helix domain containing 2 pseudogene 9	Other	Other	Anapl_01695	3,24E–03	1,53
R0LGI8	ATP5F1C	ATP synthase F1 subunit gamma	Cytoplasm	Transporter	Anapl_17212	2,85E–06	1,74
R0KXM3	SDHC	Succinate dehydrogenase complex subunit C	Cytoplasm	Enzyme	Anapl_18161	6,46E–03	1,61
**Metabolism of lipids**							
R0JHJ2	HADHA	Hydroxyacyl-CoA dehydrogenase trifunctional multienzyme complex subunit alpha	Cytoplasm	Enzyme	Anapl_17630	8,73E–07	1,67
R0JU81	CES1	carboxylesterase 1	Cytoplasm	Enzyme	Anapl_00395	3,86E–04	1,53
R0JVE7	HSD17B4	Hydroxysteroid 17-beta dehydrogenase 4	Cytoplasm	Enzyme	Anapl_07826	5,14E–03	1,79
R0L3F2	MTTP	Microsomal triglyceride transfer protein	Cytoplasm	Transporter	Anapl_12362	2,26E–06	1,55
R0L7C1	PCCB	Propionyl-CoA carboxylase subunit beta	Cytoplasm	Enzyme	PCCB	6,83E–05	2,06
U3I002	HSD17B10	Hydroxysteroid 17-beta dehydrogenase 10	Cytoplasm	Enzyme	HSD17B10	3,71E–04	1,59
U3IA20	EHHADH	Enoyl-CoA hydratase and 3-hydroxyacyl CoA dehydrogenase	Cytoplasm	Enzyme	EHHADH	1,83E–05	1,93
U3IUH7	TTPA	Alpha tocopherol transfer protein	Cytoplasm	Transporter	TTPA	1,44E–04	1,74
U3J4Z9	ACSL5	Acyl-CoA synthetase long chain family member 5	Cytoplasm	Enzyme	ACSL5	2,77E–07	1,70
U3J9A9	HMGCS1	3-Hydroxy-3-methylglutaryl-CoA synthase 1	Cytoplasm	Enzyme	HMGCS2	2,01E–04	1,57
**Metabolism of proteins**							
B2ZP77	HSPA1L	Heat shock protein family A (Hsp70) member 1 like	Cytoplasm	Other	HSPA1L	6,57E–03	1,52
R0JYD2	RAB1A	RAB1A, member RAS oncogene family	Cytoplasm	Enzyme	Anapl_07700	7,83E–04	1,66
R0LAK1	LMAN1	Lectin, mannose binding 1	Cytoplasm	Other	Anapl_12329	1,03E–04	2,85
R0LL32	SEC24B	SEC24 homolog B, COPII coat complex component	Cytoplasm	Transporter	Anapl_04790	3,76E–03	1,73
R0LXF6	RPL6	Ribosomal protein L6	Nucleus	Other	Anapl_14193	9,75E–04	1,67
S4V6M7	RPL19	Ribosomal protein L19	Cytoplasm	Other	RPL19	8,01E–03	1,64
U3IB18	YBX1	Y-box binding protein 1	Nucleus	Transcription regulator	YBX1	1,03E–04	3,46
U3IU92	ALB	Albumin	Extracellular Space	Transporter	ALB	1,11E–04	1,63
U3IX69	PTRH2	Peptidyl-tRNA hydrolase 2	Cytoplasm	Enzyme	PTRH2	8,29E–03	1,80
**Metabolism of amino acids and derivatives**							
R0JFK7	RIDA	Reactive intermediate imine deaminase A homolog	Cytoplasm	Enzyme	Anapl_03878	1,78E–03	1,66
R0L703	SFXN1	Sideroflexin 1	Cytoplasm	Transporter	Anapl_14150	7,13E–03	1,52
R0LXM2	LRRC8C	Leucine rich repeat containing 8 VRAC subunit C	Plasma Membrane	Ion channel	Anapl_10033	7,33E–03	1,57
U3IJE1	QDPR	Quinoid dihydropteridine reductase	Cytoplasm	Enzyme	QDPR	6,56E–04	1,65
**Gluconeogenesis**							
R0KUN0	PFKFB1	6-Phosphofructo-2-kinase/fructose-2,6-biphosphatase 1	Cytoplasm	Kinase	Anapl_18711	1,11E–03	1,72
R0J723	PCK2	Phosphoenolpyruvate carboxykinase 2, mitochondrial	Cytoplasm	Kinase	Anapl_18885	7,75E–03	1,69
**(B) Response to hypoxia**							
R0JIN6	PSMD7	Proteasome 26S subunit, non-ATPase 7	Cytoplasm	Other	Anapl_11901	1,12E–03	1,50
U3I0F9	PKM	Pyruvate kinase M1/2	Cytoplasm	Kinase	PKM	1,52E–05	1,56
U3I6K3	PDLIM1	PDZ and LIM domain 1	Cytoplasm	Transcription regulator	PDLIM1	1,83E–03	1,98
U3J6H9	PSMD13	Proteasome 26S subunit, non-ATPase 13	Cytoplasm	Peptidase	PSMD13	1,75E–04	2,69
**Apoptosis**							
A0A076G731	MAPK1	Mitogen-activated protein kinase 1	Cytoplasm	Kinase	MAPK1	1,53E–04	1,59
U3IFN5	VIL1	Villin 1	Cytoplasm	Other	VIL1	5,56E–03	3,81
U3IRY0	VIM	Vimentin	Cytoplasm	Other	VIM	3,64E–03	1,82
**Metabolism of proteins**							
R0JSL2	XPNPEP3	X-prolyl aminopeptidase 3	Cytoplasm	Peptidase	Anapl_16747	3,74E–03	1,57
R0JIN6	PSMD7	Proteasome 26S subunit, non-ATPase 7	Cytoplasm	Other	Anapl_11901	1,12E–03	1,50
R0LNX2	UBA3	Ubiquitin like modifier activating enzyme 3	Cytoplasm	Enzyme	Anapl_03595	7,72E–03	1,52
U3J6H9	PSMD13	Proteasome 26S subunit, non-ATPase 13	Cytoplasm	Peptidase	PSMD13	1,75E–04	2,69
R0JE49	EIF4G3	Eukaryotic translation initiation factor 4 gamma 3	Cytoplasm	Translation regulator	Anapl_13058	9,07E–03	1,59
U3IJM3	KPNA4	Karyopherin subunit alpha 4	Nucleus	Transporter	KPNA4	2,01E–04	4,22
R0JC74	AP1G1	Adaptor related protein complex 1 subunit gamma 1	Cytoplasm	Other	Anapl_08470	2,78E–03	1,82
U3ICK1	ARCN1	Archain 1	Cytoplasm	Other	ARCN1	3,08E–03	2,53
R0LVK1	TOM1	Target of myb1 membrane trafficking protein	Cytoplasm	Transporter	Anapl_09649	2,59E–03	1,53
R0KLF1	RAB14	RAB14, member RAS oncogene family	Cytoplasm	Enzyme	Anapl_16651	5,40E–04	2,30
**Cytoskeleton organization**							
R0JL69	PDLIM5	PDZ and LIM domain 5	Cytoplasm	Other	Anapl_07981	1,83E–03	3,68
R0JUU9	CKAP4	Cytoskeleton associated protein 4	Cytoplasm	Other	Anapl_14117	2,45E–04	3,18
R0KCF6	TES	Testin LIM domain protein	Plasma membrane	Other	Anapl_06809	2,24E–03	1,72
R0L5A5	TPM1	Tropomyosin 1	Cytoplasm	Other	Anapl_02698	8,69E–03	1,62
R0LM85	MYLK	Myosin light chain kinase	Cytoplasm	Kinase	Anapl_00914	5,92E–03	1,87
R0M8F1	GLG1	Golgi glycoprotein 1	Cytoplasm	Other	Anapl_07485	1,94E–05	1,65
R4HH66	DES	Desmin	Cytoplasm	Other	NA	8,58E–04	1,81
U3I6K3	PDLIM1	PDZ and LIM domain 1	Cytoplasm	Transcription regulator	PDLIM1	1,83E–03	1,98
U3I9S0	TPM2	Tropomyosin 2	Other	Other	TPM2	3,86E–04	2,52
U3IFN5	VIL1	Villin 1	Cytoplasm	Other	VIL1	5,56E–03	3,81
**Glycolytic process**							
U3I0F9	PKM	Pyruvate kinase M1/2	Cytoplasm	Kinase	PKM	1,52E–05	1,56
R0JH21	OGDHL	Oxoglutarate dehydrogenase like	Other	Enzyme	OGDHL	5,43E–03	2,55
**Extracellular matrix organization**							
R0JJW5	COL6A2	Collagen type VI alpha 2 chain	Extracellular space	Other	Anapl_09440	3,18E–03	1,62
U3ID88	COL6A3	Collagen type VI alpha 3 chain	Extracellular space	Other	COL6A3	1,83E–03	2,05
**Oxidation-reduction process**							
R0JH21	OGDHL	Oxoglutarate dehydrogenase like	Other	Enzyme	OGDHL	5,43E–03	2,55
R0K4Q9	P3H1	Prolyl 3-hydroxylase 1	Nucleus	Enzyme	Anapl_10218	7,67E–04	1,65
R0JQV4	PTGR2	Prostaglandin reductase 2	Cytoplasm	Enzyme	Anapl_01128	5,56E–03	2,89

*Pathways were retrieved using the ProteInside sofware and only biological process with a *p*-value < 10^–3^ and a number of variant proteins involved ≥ 2 were represented.*

**FIGURE 3:**
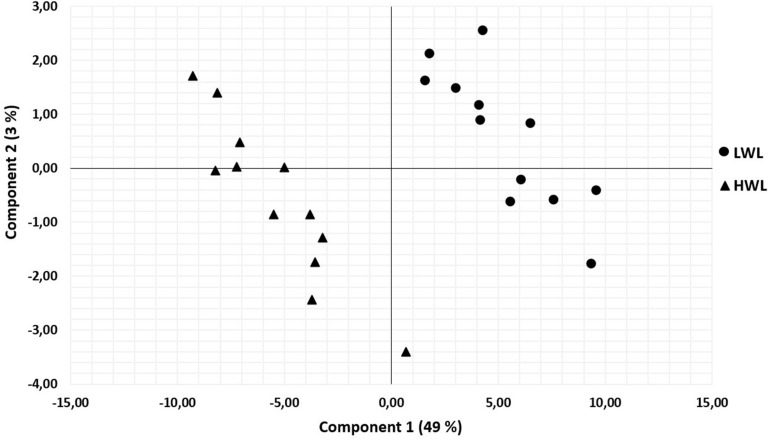
Plot of the two first principal component score vectors showing variability according to the two liver weight groups for the 78 variant proteins (*n* = 12/groups) : LWL = liver weight < 600 g = ∙, HWL = liver weight > 700 g = ▲.

Finally, the PLS-DA analysis showed that in order to discriminate the 2 liver weight groups, 68 proteins were identified with a VIP > 0.8: 37 of them were overexpressed in the low weight livers and 31 ones were overexpressed in the high weight livers (Figure 3). The identified proteins are related to several pathways associated with energy metabolism, metabolism of lipids or proteins, cytoskeletal organization, cellular death by apoptosis, carbohydrates metabolism, metabolism of amino acids and their derivatives and extracellular matrix organization.

### Energy Metabolism

We observed in low weight livers, the overexpression of proteins such as NADH:ubiquinone oxidoreductase (NDUFA4, NDUFA2, NDUFB6, NDUFV1, NDUFA9) enzymes that are parts of the mitochondrial respiratory chain complex I assembly. The succinate dehydrogenase, involved in both the tricarboxylic acid cycle and the electron transport chain and the ATP synthase F1 subunit gamma enzyme from the complex V of mitochondrial respiratory chain were also overexpressed in low weight livers. In high weight livers, the overexpression of proteasome 26S subunit, protein pyruvate kinase (PKM) and oxoglutarate dehydrogenase like (OGDHL) was observed. It was also noticed an overexpression of PDZ and LIM domain protein 1 in high weight livers.

### Metabolism of Lipids

In low weight livers, the ovexpression of proteins such as 3-hydroxy-3-methylglutaryl coenzyme synthase and acyl-CoA synthetase long chain family member 5 was noticed. We also remarked the overexpression of carboxylesterase 1, propionyl-CoA carboxylase beta chain, mitochondrial enoyl-CoA hydratase, hydroxyacyl CoA dehydrogenase and hydroxysteroid 17-beta dehydrogenase. Finally, we also observed the overexpression of the Microsomal Triglyceride Transfer Protein (MTTP) and the Alpha-Tocopherol Transfer Protein (TTPA) in samples from the LWL group.

### Metabolism of Proteins

In low weight livers, proteins such as Ribosomal proteins, Ras-related protein Rab-1A and transport protein Sec24B (component of the coat protein complex II- COPII) were overexpressed. On the contrary, proteins such as ubiquitin-like protein NEDD8 (UBA3), proteasome 26S subunit, karyopherin subunit alpha 4 (KPNA4) and adaptor related protein complex 1 subunit gamma 1 (AP1G1) were overexpressed in high weight livers.

### Cytoskeleton Organization

Cytoskeleton related proteins, PDZ and LIM domain protein 1/5 (PDLIM1/5), testin LIM domain protein, tropomyosin 2 and Golgi glycoprotein 1 or Golgi apparatus protein 1 were overexpressed in high weight livers.

### Programmed Cell Death

In high weight livers, an increase in vimentin and mitogen-activated protein kinase 1 contents was observed. In those livers, the overexpression of OGDHL was also noticed.

### Others Metabolic Pathways

Among the overexpressed proteins in LWL, some of them are the reactive intermediate imine deaminase, the sideroflexin 1 (a mitochondrial transporter of amino acids), the dihydropteridine reductase and the leucine rich repeat containing eight VRAC subunit C. On the contrary, in HWL, the overexpression of prolyl 3-hydroxylase, a collagen type VI alpha 2 chain (COL6A2) and collagen type VI alpha 3 chain (COL6A3) was found.

## Discussion

During the overfeeding period, the hepatic steatosis develops with a huge accumulation of triglycerides in hepatocytes and consequently the weight of the liver of overfed ducks rises from about 80 to more than 500 g (Théron, 2011; Rémignon et al., 2018; Bonnefont et al., 2019). As described by Locsmándi et al. (2007), in steatotic duck livers, lipids are stored in macrovacuoles located in the cytoplasm of hepatocytes. Hermier et al. (1999) showed that, during the overfeeding period, the lipids content of livers rises from 5% to more than 50% of the dry matter (DM). In the present study, livers from the two groups (LWL and HWL) also presented a high percentage of lipids (approximately 57% of DM).

### Energy Metabolism

In *in vitro* culture of mammalian preimplantation embryos, Koerber et al. (1998) showed an overexpression of the NADH:ubiquinone oxidoreductases in samples with a higher oxygen concentration. Conn et al. (2001) also demonstrated that a decrease in the NADH:ubiquinone oxidoreductase activity is associated with an increase in the oxidative stress level in Parkinson’s disease. García-Aguilar and Cuezva (2018) reported that the increased expression of the ATP synthase allows the cell to be less exposed to the oxidative stress. These results therefore show that higher oxygen concentration might be found in low weight livers, while in high weight livers the level of oxidative stress seems to be higher. However, according to García-Aguilar and Cuezva (2018), in the liver, the inhibition of the ATP synthase leads to reprogramming energy metabolism into enhanced glycolysis. Indeed, PKM is the enzyme that catalyzes the rate-limiting step of glycolysis generating pyruvate and ATP (Israelsen and Vander Heiden, 2015). Then, high weight livers have a more active glycolysis pathway than low weight livers. Moreover, Sen et al. (2012) reported that in humans, OGDHL, one isoform of the protein oxoglutarate dehydrogenase (OGDH), degrades glucose and glutamate. According to Groulx and Lee (2002) and Abu-El-Rub et al. (2019), the proteasome 26S subunit was directly involved in the cellular response to hypoxia by degrading hypoxia inducible factor (HIF) via oxygen-dependent proline hydroxylation. Luo et al. (2011) reported that the pyruvate kinase 2 (PKM) isoform is activated by the hypoxia-inducible factor 1 (HIF-1) and promotes the transactivation of HIF-1 target genes by enhancing HIF-1 binding and recruitment of hypoxia response elements. However, in our study, it was not possible to distinguish if one or both isoforms of PKM were present. Finally, Ishikawa et al. (2010) showed that, in mice, by comparing gene expression profile of hyperoxic and hypoxic retinas, the PDZ and LIM domain (PDLIM1) proteins genes were under-expressed in hyperoxic retinas.

During overfeeding, excessive energy intake results in an increase in liver weight. This leads on the one hand to a physical obstruction and on the other hand to an increase in blood lipemia (Blum et al., 1970) resulting in a decrease in the partial pressure of oxygen in the fatty liver. In addition, the high lipid content in HWL indicates that lipids synthesis remains very important. Consequently, the lipid oxidation is also very active and this oxidation consumes oxygen, which could also contribute to the oxygen depletion of this organ. This may be related to all our results which suggest that a more aerobic respiration and therefore a higher oxygen partial pressure is present in low weight livers in contrast with high weight livers which are more characterized by hypoxia and oxidative stress.

### Metabolism of Lipids

At the end of the overfeeding period, the differentially expressed proteins involved in lipid metabolism can be further assigned to three pathways : biosynthesis, beta-oxidation, or transport of lipids. Clinkenbeard et al. (1975) proposed that the cytoplasmic 3-hydroxy-3-methylglutaryl-CoA synthase, involved in step 2 of the subpathway that synthesizes (R)-mevalonate from acetyl-CoA, also acts in cholesterogenesis in the liver. According to Xu et al. (2014) carboxylesterase 1 is an enzyme that hydrolyses triglycerides and cholesterol esters and is important for lipids metabolism. According to Suzuki et al. (1990), the level of long-chain acyl-CoA synthetase mRNA is increased 7 to 8-fold in rat liver by feeding a diet high in carbohydrates or fat, which is consistent with the physiological significance of that enzyme in fatty acids metabolism. According to Parkes et al. (2006), the storage of fatty acids as tryglycerides needs the acyl-CoA synthetase (ACSL) to activate fatty acids in long-chain acyl-CoAs (LCA-CoAs). Therefore, our results show that the hepatocytes seem to adapt to liver steatosis development by decreasing their capacity for lipid anabolism for not ending in a pathological state. However, the LCA-CoAs can also enter the β-oxidation pathway for energy production. Then, long-chain acyl-CoA synthetase catalyzes the conversion of long-chain fatty acids to their active form, i.e., acyl-CoAs, for both the synthesis of cellular lipids and their degradation via beta-oxidation (Nakahara et al., 2012; Ohkuni et al., 2013). Kalousek et al. (1980) showed that the Propionyl-CoA carboxylase beta chain is an enzyme involved in the catabolism of odd chain fatty acids while enoyl-CoA hydratase and hydroxyacyl CoA dehydrogenase are respectively the second and penultimate enzymes directly involved in the beta-oxidation pathway. Several previous studies reported that the hydroxysteroid 17-beta dehydrogenase is a bifunctional enzyme acting on the peroxisomal beta-oxidation pathway of fatty acids (Prehn et al., 2009). Consequently, in the present study, we can assume that in the LWL group, the activity of the beta-oxidation pathway was reduced. These results are in agreement with those described by Hérault et al. (2010) who showed a negative effect of feeding on the activity of beta-oxidation genes. The level of MTTP largely differs between the two liver weight groups. MTTP is an enzyme that transfers triglycerides to nascent apolipoproteins B for producing VLDL and finally participates in removing lipids out from the hepatocyte. In overfed ducks, Hérault et al. (2010) showed a negative correlation between MTTP mRNA level and liver weight (*r* = −0.23). Lim and Traber (2007) showed that alpha-tocopherol transfer protein (TTPA) is a liver cytosolic transport protein that facilitates alpha-tocopherol transfer into liver secreted plasma lipoproteins. Then, as a whole, these results suggest a more active lipid metabolism through higher export and beta-oxidation levels in the LWL group compared to the HWL one. Zhu et al. (2011) also reported that in the liver tissue of geese, overfeeding involves upregulation of genes playing important roles in lipogenesis and TG synthesis, and a downregulation of genes involved in glycolysis, cholesterol metabolism and oxidation. According to Hermier et al. (1994) and Fournier et al. (1997), the complex interplay of beta-oxidation of fatty acids and tryglycerides secretion caused the differences of liver steatosis ability between Pekin and Muscovy ducks. These observations could also be connected with previous observations regarding the hypoxic level of the two livers weights groups. According to Rankin et al. (2009), a severe hypoxia promotes lipids accumulation mainly by preventing mitochondrial β-oxidation and by promoting lipids droplets formation. In contrast, a light hypoxia suppresses lipids accumulation by inhibiting *de novo* lipogenesis but also by promoting lipids storage and exports.

### Metabolism of Proteins

The amount of total protein differs according to liver weights despite a similar lipid and dry matter contents. According to Green and Lorsch (2002) and Klein et al. (2004), ribosomal proteins are involved in peptide chains elongation and eukaryotic translation terminations. According to Zhou et al. (2015), those proteins are primarily responsible for protein synthesis. Several studies showed that the coat protein complex II (COPII) is involved in transporting endoplasmic reticulum (ER) membrane proteins into vesicles and in the selection of cargo molecules for transport to the Golgi (Mancias and Goldberg, 2008). However, according to Bohnsack and Haas (2003), in humans, neddylation is mediated by UBA3 and Osaka et al. (1998) reported that this is associated with proteolysis. According to Bard et al. (2018), proteasome 26S is the principal proteolytic machine responsible for regulated protein degradations in eukaryotic cells.

The low weight livers could therefore be characterized by more anabolism than catabolism of proteins in opposition to what is observed in high weight livers. Bénard and Labie (1998) reported a 2.5-fold change in liver proteins quantity only during the first half of the overfeeding period. According to Bax et al. (2012), during that period, hepatocytes must synthesize the enzymes necessary to metabolize the huge quantity of glucose molecules coming from corn (starch) digestion. However, according to Wanninger et al. (2011), there is an increase of the proteolytic activity during the development of the non-alcoholic steato-hepatitis to counterbalance a possible development of fibrosis associated with the dysfunctionality of the liver. On the contrary, Awde et al. (2013) reported a decrease of proteolytic activities in livers during the period of overfeeding in ducks. Those authors finally suggested that proteases reduce their activities probably to facilitate the cellular adaptation to the increase of the level of energy metabolism. However, it is noticeable that their liver weights range was only similar to the one observed in our LWL group. On the contrary, Rémignon et al. (2018), from liver weights values similar to the ones observed in our HWL group, suggested that the metabolism of the high weight livers was more oriented toward the prevention of a fibrosis development. Therefore, we can imagine that up to a certain liver weight, the proteins proteolysis level remains low to facilitate the cellular adaptation induced by the overfeeding. On the contrary, if the liver weight reaches a high value, as a consequence of the very high development of the steatosis, then the proteolytic activities increase to prevent a fibrosis development but also to provide energy or to face to the high turnover of metabolic enzymes This increased catabolism in high weight livers could also explains the decrease in total proteins content observed in the livers of the HWL group.

### Cytoskeleton Organization

According to Kadrmas and Beckerle (2004), LIM proteins are involved in many signaling pathways due to their location in the nuclear and cytoskeletal compartments. They also regulate gene expression to affect cytoskeletal dynamics and vice versa. LIM proteins are also markers of increased size of hepatocytes (Ng et al., 2011). Mölleken et al. (2009) showed that the expression of tropomyosin was significantly higher (52.4-fold increase) in cirrhotic septa. Matsuda et al. (1991) reported that the secretion of hepatic glycoproteins in the Golgi apparatus were impaired in alcoholic liver injury. Then, according to Otogawa et al. (2008), in rat liver tissue, an increased expression of tropomyosin was observed throughout the fibrosis development and they suggested that this could be used as a diagnostic marker of liver fibrosis. Consequently, in the present study, all the observed modifications of the proteins related to the cytoskeleton could simply reflects an adaptation to the lipidic overload which is particuarly important in the livers of the HWL group.

### Programmed Cell Death

According to Byun et al. (2001), the proteolysis of vimentin by caspases promotes apoptosis by dismantling intermediate filaments and amplifying the cell death signal via a pro-apoptotic cleavage product. Associated with mitogen-activated protein kinase 1, the overexpression of this protein confirms the activation of programmed cell death by apoptosis in HWL group (Zhu et al., 1999; Byun et al., 2001). In addition, according to Sen et al. (2012), the increased expression of OGDHL increased the production of reactive oxygen species (ROS) which led to apoptosis through a negative regulation of the AKT signaling cascade by caspase 3 and a decreased phosphorylation of NF-κB. Then, our results could suggest a more important apoptotic activity in high weight livers. According to Alkhouri et al. (2011), an increase of apoptosis is typically present in patients with NAFLD and in experimental models of steatohepatitis. Kanda et al. (2018) also reported that apoptosis seems very important in the progression of NAFLD/NASH. In duck fatty livers, Rémignon et al. (2018) reported that, at the end of the overfeeding period, the level of apoptosis increased and they suggested that it is probably due to the fact that some hepatocytes are no longer able to support the very high level of metabolism induced by the overfeeding.

### Others Metabolic Pathways

The reactive intermediate imine deaminase, the sideroflexin 1 (a mitochondrial transporter of amino acids), the dihydropteridine reductase and the leucine rich repeat containing eight VRAC subunit C are related to the metabolism of amino acids and their derivative (Lutter et al., 2017). According to Hudson and Eyre (2013), prolyl 3-hydroxylation is a rare but conserved post-translational modification in many collagen types. In addition, according to Stickel et al. (2001), the amount of collagen VI was elevated in all livers from alcoholic patients compared to controls, so it appears to be a sensitive marker indicating a fibrotic transformation in alcoholics liver steatosis. These results are in accordance with the observations reported by Veidal et al. (2011) who showed that a test for MMP-degraded type VI collagen was strongly associated with hepatic fibrosis development in two animal models. However, histological studies performed on liver of overfed mule ducks showed only cellular hypertrophy and no evidence of membrane damage or fibrosis at the end of the overfeeding period (Hermier et al., 1999; Locsmándi et al., 2007; Théron, 2011). Then, it must be concluded that the steatotic liver of overfed ducks mainly synthesizes larger amounts of connective tissue just to support the increase in weight.

## Conclusion

The present study demonstrated that at the end of the overfeeding period, the proteomic profiles of duck livers differ according to the liver weight class (550 g < < 600 g vs. > 700 g). As summarized in Table 2, low weight livers (550 < < 600 g) are characterized by a greater anabolism of proteins and a larger export and beta-oxidation of lipids than in high weight livers (>700 g). On the contrary, in HWL, the massive storage of lipids caused a reorganization of the cytoskeleton. In LWL the aerobic conditions still allow the synthesis of energy in ATP form via the electron transport chain while in HWL, the larger storage of lipids and the reduction in the amount of proteins accompany cellular responses to hypoxia, an increase in ROS production and cytoskeleton modifications and apoptosis. Therefore, it seems that hepatocytes from ducks are more or less prone to adapt to the metabolic pressure imposed by overfeeding. Based on the highest value of ratio, the level of NADH:ubiquinone oxidoreductase, Karyopherin subunit alpha 4, LIM protein, Vilin 1, Y-box binding protein 1 and Propionyl-CoA could be relevant to select ducks having the highest ability for “foie gras” production. In the future, more interests could be given to the identification, as early as possible, of such birds more or less adapted to easily support overfeeding.

**TABLE 2:** Summary of main cellular characteristics differing between high and low weight livers.

Low weight livers : 550–600 g	High weight livers : > 700 g
Lipids export and beta-oxidation : increased	Lipid storage : very high
Respiratory electron transport and ATP synthesis : maintained	Oxidative stress: increased
Protein anabolism : increased	Catabolism of proteins : increased
	Cytoskeletal and extracellular matrix : important re-organization
	Apoptosis : increased

## Data Availability Statement

The mass spectrometry proteomics data have been deposited to the ProteomeXchange Consortium via the PRIDE partner repository with the dataset identifier PXD019866.

## Ethics Statement

Ethical review and approval was not required for the animal study because we collected samples from a commercial slaughther-house after the regular operations of anesthesia, bleeding, scalding, plucking, and evisceration. We do not manipulate live animals and no specific experimental manipulations were performed during the time of rearing. All the samples we harvested were commercial ones, ready to be purchased by consumers. Consequently, we do not need any ethical review and approval. However, we indicate within the manuscript (L 299-303) that investigators were certificated by the French governmental authority (agreement no. R-31-ENVT-F1-12) and Federation of European Laboratory Animal Science Associations (agreement no. F011/05) for carrying out those experiments.

## Author Contributions

BL, HR, and RD designed the study. NM-G, CP, CB, HR, and BL contributed in sample collection and analysis. CP and NM-G conducted LC/MS/MS analysis and CP protein identification and quantification. BL performed the statistical and GO analysis. BL analyzed and interpreted all results with the help of HR, NM-G, and CP. CP wrote proteomics analysis sections and BL wrote the remaining sections of the manuscript. All authors read but BL and HR approved the submitted version.

## Conflict of Interest

RD was employed by company Idena. The remaining authors declare that the research was conducted in the absence of any commercial or financial relationships that could be construed as a potential conflict of interest.

## Footnotes

1 https://github.com/mzdb

2 http://matrixscience.com

3 http://proline.profiproteomics.fr/
